# Identification of an extensive gene cluster among a family of PPOs in *Trifolium pratense *L. (red clover) using a large insert BAC library

**DOI:** 10.1186/1471-2229-9-94

**Published:** 2009-07-20

**Authors:** Ana Winters, Sue Heywood, Kerrie Farrar, Iain Donnison, Ann Thomas, K Judith Webb

**Affiliations:** 1 Institute of Biological, Environmental and Rural Sciences, Aberystwyth University, Gogerddan, Aberystwyth, Ceredigion, SY23 3EB, UK; 2 CNAP Artemisia Research Project, Department of Biology – Area 7, University of York, Heslington, PO Box 373, York, YO10 5YW, UK

## Abstract

**Background:**

Polyphenol oxidase (PPO) activity in plants is a trait with potential economic, agricultural and environmental impact. In relation to the food industry, PPO-induced browning causes unacceptable discolouration in fruit and vegetables: from an agriculture perspective, PPO can protect plants against pathogens and environmental stress, improve ruminant growth by increasing nitrogen absorption and decreasing nitrogen loss to the environment through the animal's urine. The high PPO legume, red clover, has a significant economic and environmental role in sustaining low-input organic and conventional farms. Molecular markers for a range of important agricultural traits are being developed for red clover and improved knowledge of PPO genes and their structure will facilitate molecular breeding.

**Results:**

A bacterial artificial chromosome (BAC) library comprising 26,016 BAC clones with an average 135 Kb insert size, was constructed from *Trifolium pratense *L. (red clover), a diploid legume with a haploid genome size of 440–637 Mb. Library coverage of 6–8 genome equivalents ensured good representation of genes: the library was screened for polyphenol oxidase (PPO) genes.

Two single copy PPO genes, PPO4 and PPO5, were identified to add to a family of three, previously reported, paralogous genes (PPO1–PPO3). Multiple PPO1 copies were identified and characterised revealing a subfamily comprising three variants PPO1/2, PPO1/4 and PPO1/5. Six PPO genes clustered within the genome: four separate BAC clones could be assembled onto a predicted 190–510 Kb single BAC contig.

**Conclusion:**

A PPO gene family in red clover resides as a cluster of at least 6 genes. Three of these genes have high homology, suggesting a more recent evolutionary event. This PPO cluster covers a longer region of the genome than clusters detected in rice or previously reported in tomato. Full-length coding sequences from PPO4, PPO5, PPO1/5 and PPO1/4 will facilitate functional studies and provide genetic markers for plant breeding.

## Background

Polyphenol oxidases (PPOs) are implicated in a range of biological functions in diverse systems. In addition to a role in black/brown pigment biosynthesis, PPOs may also have protective roles in plants against pathogens and environmental stress. While PPO-induced browning is a major problem in the food industry, causing massive losses through unacceptable discolouration in fruit and vegetables [1,2], it is also implicated in plant defence against bacterial and fungal diseases of diverse plant species [3-7]. Down-regulating constitutive and induced expression of PPOs in tomato by antisense methods resulted in increased pathogen susceptibility [7]. In the forage legume *Trifolium pratense *L. (red clover), PPO activity also provides some protection against natural infestations of sciarid fly, thrips and aphids under semi-controlled conditions [8].

PPO activity in red clover is an agriculturally and environmentally important trait. Red clover provides a significant and sustainable component of grazed pastures in low-input organic and conventional farms and is harvested for conservation as hay or silage in Europe and North America [9]. Major nutritional benefits of PPO activity have been recognised in this crop; high levels of PPO activity confer protection against protein degradation by micro-organisms in the animal rumen [10,11] and by plant enzymes during ensilage [12,13]. Lower protein degradation in the rumen and during ensiling results in increased nitrogen absorption by ruminants and simultaneously decreases nitrogen loss to the environment through the animal's urine.

PPO enzymes are ubiquitous and found in a broad range of dicotyledonous and monocotyledonous species. In legumes only a latent form of PPO enzyme was reported in leaves of the grain legume, *Vicia faba *[14], but active PPO enzymes are constitutively expressed in both aerial and root tissues in *T. pratense*. Thus, *T. pratense *offers an ideal opportunity to study a PPO gene family and aspects of PPO function. Complete coding sequences, but not promoter regions, of PPO genes PPO1, PPO2 and PPO3, have previously been reported [15]. Expression patterns of the three known PPO genes vary in red clover: PPO1 is most abundant in young leaves, PPO2 in flowers and petioles, and PPO3 in leaves and also possibly in flowers [15]. In tomato (*Lycopersicon esculentum *Mill.), expression profiles of a six-member PPO gene family (PPOs A/A', B, C, D, E and F) revealed differential PPO expression [7,16]. PPO B is highly expressed in young tomato leaves, whereas transcripts of PPO B, E and F dominate in the inflorescence. Specific PPO transcripts are also associated with different trichome types.

The tomato PPO gene family has six paralogous genes, which all appear to be clustered on a 165 Kb region on chromosome 8 [17]. The genomic relationship between members of the *T. pratense *PPO gene family is unknown, but similarities in gene structure and function, combined with differences in individual PPO gene expression profiles in red clover [15], suggest that these red clover PPO genes are also paralogues. Such gene duplication, followed by divergence from the parent sequence by mutation and selection or drift, is believed to provide a platform for evolutionary change within genomes [18].

The haploid genome size of *T. pratense *has previously been estimated as 637 Mb when measured by microdensitometry of Feulgen-stained nuclei [19] and, more recently, as 440 Mb when measured by flow cytometry [20]. Two red clover libraries already exist [20] but they have relatively small insert sizes. Here, we describe the creation of a new *T. pratense *BAC library with a larger insert size and its use in isolating additional PPO genes and their regulatory regions and in determining the relationship between PPO gene family members within the *T. pratense *genome.

## Results

### BAC library construction and validation

**Figure 1:**
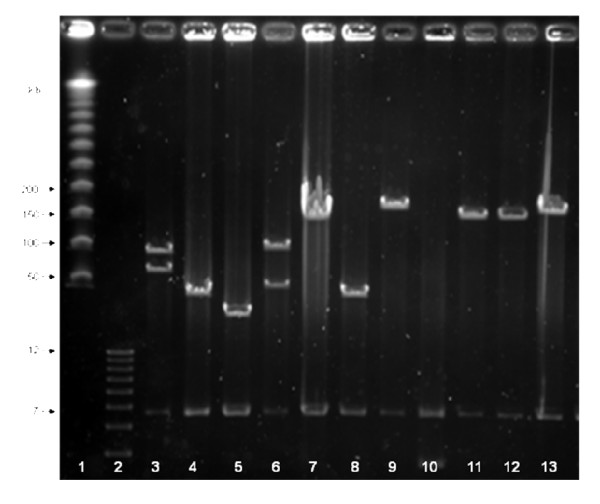
***T. pratense *inserts released by digestion from 58 randomly selected BAC clones**. Using *Not*1, DNA was separated by pulse-field gel electrophoresis (PFGE). BACs were generated by restricting *T. pratense *gDNA with *Hind*III, PFGE and cloning the size separated gDNA in the size region of 150–100 Kb. Molecular weight standards are lane 1, lambda ladder (NEB, Beverley, Mass., USA) and lane 2, DNA Molecular Weight Marker X (Roche); pIndigoBAC5 *Not*I vector fragment is 7 Kb. The average insert size calculated from all 11 BAC clones in lanes 3–13 is estimated as 113 Kb.

**Figure 2:**
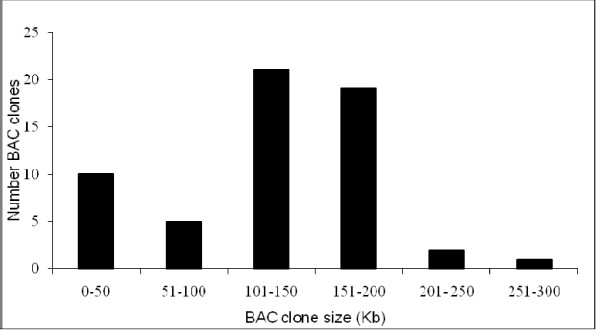
**Distribution of DNA insert size of 58 *T. pratense *BAC clones**. Insert sizes in Kb were calculated from *Not *1 digests of BAC DNA following fractionation by pulse-field gel electrophoresis. The average insert size of the library was estimated at 135 Kb.

The *T. pratense *BAC library was constructed from partially digested gDNA in a single, high molecular weight, size selection experiment. A total of 26,016 BACs were picked into 271 96-well plates, with an estimated average insert size of 135 Kb per BAC clone, based on 58 randomly selected BAC clones (Figure 1, 2).

### PCR-based screen of BAC library and PPO sequence analysis

The primer pairs specific to PPO2, PPO4 and PPO5 identified 5–6 BACs each, indicating one copy of each gene. By contrast, the PPO1 primer pair identified at least 28 BAC clones (Table 1). All PPO genes were sequenced directly from selected BAC clones. An iterative process of sequencing and primer design revealed a subfamily of PPO1.

**Table 1:** Number of estimated BAC clones, confirmed sequences and predicted copy number of members of the PPO gene family identified in a *T. pratense *BAC library

Gene	PPO variant	Estimated no. BAC clones containing PPO	Confirmed no. sequences from BAC clones	Predicted PPO copy no.
PPO1 (total)		≥ 28	11	3–5

	PPO1/2	4	2	1

	PPO1/4	≥ 26^a^	5	1

	PPO1/5	9	4	1–2

PPO2		5	1	1

PPO4		6	1	1

PPO5		5	2	1

^a^20/47 PCR products from gDNA superpools of BAC library were sequenced and confirmed as PPO1/4.

**Figure 3:**
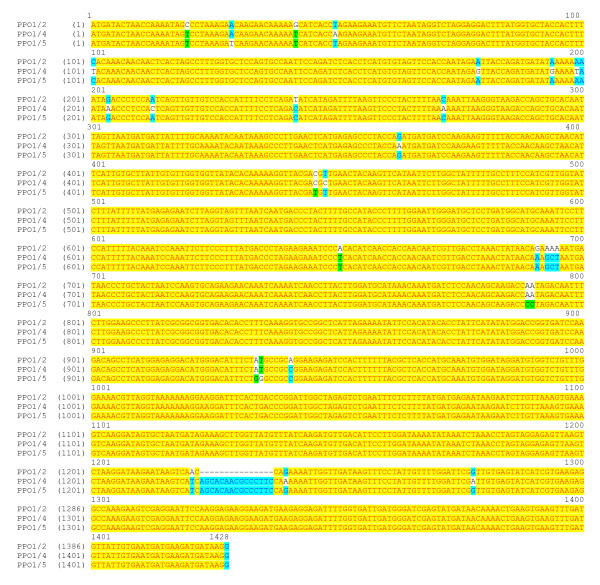
**DNA sequence alignment of three variants of PPO1 gene isolated from *T. pratense***. PPO1/2 is a partial sequence; PPO1/4 is complete coding region [GenBank:FJ587214]; PPO1/5 is complete coding region and most similar to published PPO1 [GenBank:AY017302]. The figure was generated in Vector NTI and formatted in word.

Three variants PPO1/2, PPO1/4 and PPO1/5 could be clearly distinguished based on their coding regions (Figure 3) and were further distinguished by differences in their flanking sequences. Primer pairs specific to variants PPO1/2 and PPO1/5 initially identified four and nine BAC clones, respectively (Table 1). In contrast, at least 26 BAC clones with PPO1/4 were identified from the PCR-based screen of the BAC library (Table 1).

Sequencing confirmed the presence of PPO1/2 on two BAC clones and PPO1/5 on four BAC clones. Five of the 26 BAC clones harbouring PPO1/4 were analysed further. Three of the five BACs also harboured other PPO genes, while the remaining two contained PPO1/4 alone; BAC-end sequencing showed homology regions with fully sequenced BAC 212 G7, indicating that the solitary PPO1/4 gene resided within this larger BAC clone.

Further sequence analysis of PPO1/5 revealed that one of the four BAC clones contained a 100 bp deletion in 1.7 Kb of 3' non-coding flanking region; otherwise there was >99.5% identity in both PPO coding and flanking sequences, differing only in six separate, single bases. PPO1/5 has the highest homology (99%) with the previously reported PPO1 [15].

### Sequence analysis of PPO4 and PPO5

**Figure 4:**
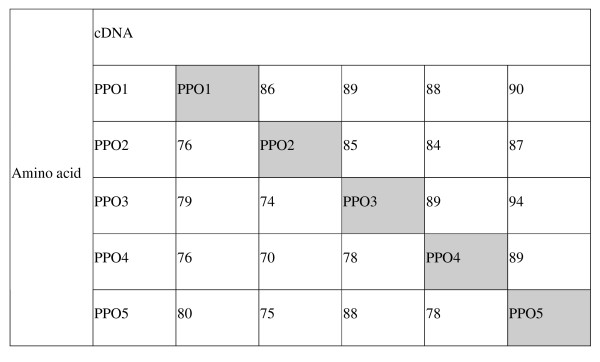
**Red clover PPO identities at the cDNA and amino acid levels**.

Full length coding DNA sequences of PPO4 [GenBank: EF183483.1] and PPO5 [GenBank: EF183484.1] were deduced from BAC sequences; neither gene contained introns. PPO4 and PPO5 sequences encode predicted proteins comprising 604 and 605 amino acids with molecular weights of 68.4 and 68.6 kDa, respectively. Identity between PPO1, PPO2, PPO3, PPO4 and PPO5 genes at the cDNA and amino acid sequence levels are 84–94% and 70–88%, respectively, with PPO3 and PPO5 showing highest homology (Figure 4). Flanking DNA sequences show little homology, indicating that the PPO genes are in different positions on the genome and therefore verify their separate identities (Table 1).

### PPO gene clusters

**Figure 5:**
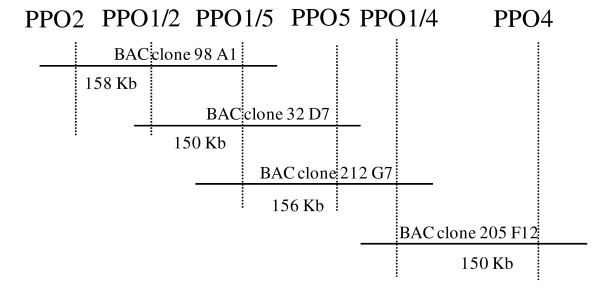
**Diagram of cluster of 6 PPO genes detected on four separate BAC clones**. The four BAC clones have been aligned based on detection of specific PPO genes by PCR; the cluster is estimated to span a maximum of 510 Kb.

Some BAC clones contained more than one PPO gene and this information was used to create a map of a predicted PPO cluster (Figure 5). For example, out of five separate BAC clones containing PPO1, one contained PPO1/5 alone (BAC 52 A5), a second contained PPO2, PPO1/2 and PPO1/5 (BAC 98 A1), a third contained PPO1/2, PPO1/5 and PPO5 (BAC 32 D7), a fourth contained PPO1/4, PPO1/5 and PPO5 (BAC 212 G7), and a fifth contained PPO1/4 and PPO4 (BAC 205 F12). Analysis of four of these BAC clones containing 11 identified PPO genes provided evidence of a potential cluster of six distinct PPO genes within 190–510 Kb (Figure 5). The full sequence of BAC 212 G7 confirmed the presence of three PPO genes (PPO1/5, PPO5 and PPO1/4) and no other plant genes; however, retrotransposons were detected. The minimum PPO cluster length is based on 156,267 bp of sequence from BAC clone 212 G7 plus sequence from PPO2, PPO1/2 and PPO4 genes and their flanking regions and a calculation of sequence overlap between BAC clones 205 F12 and 32 D7 with 212 G7.

Alignment of sequenced BAC 212 G7 and BAC 52 A5, containing the single copy of PPO1/5, revealed about 1.5 Kb identical flanking sequences; in addition, M13 (-20) derived BAC-end sequence of BAC 52 A5 was contained within BAC 212 G7, indicating that this PPO gene also lies within the proposed gene cluster.

PPO3 has not been identified in this red clover BAC library. However, both PPO3 and PPO5 have been detected by sequencing PCR products of individual plants from cultivars Sabtoron, Britta and Milvus, including the genotype used to generate the BAC library, using diagnostic primers. Coding regions of PPO3 and PPO5 differ (88% amino acids and 94% DNA; Figure 4), but show 98% homology over 171 bp of 3' flanking region.

**Figure 6:**
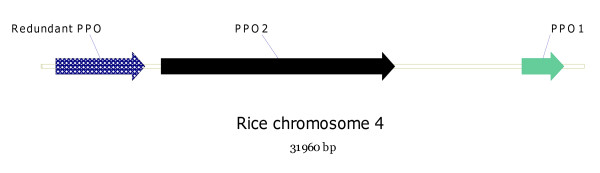
**Schematic representation of PPO gene cluster in rice taken from rice chromosome 4 [GenBank: **AP008210.1 31754771–31786730**]**. PPO1: [GenBank:AK108237.1] (DNA), [PDB:CAE03510.2] (amino acid); PPO2: [PDB:CAH66801.1] (amino acid).

A search of the GenBank database revealed that rice has two PPO genes in tandem on a 29,943 bp sequence [GenBank: AP008210] (Figure 6), with at least one of these rice PPO genes being expressed [GenBank: NM_001060467.1]. In *Medicago truncatula *[GenBank: AC157507.2] there are two PPOs, which differ by 11%, on an 8 Kb genomic sequence, but no equivalent ESTs have yet been deposited in the databases.

### Relationship of DNA sequences of PPO

**Figure 7:**
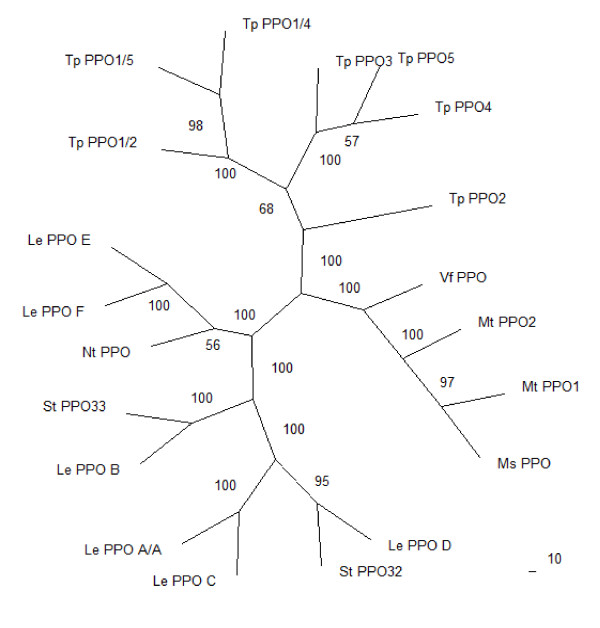
**Phylogenetic tree of coding DNA sequences of selected PPO genes and gene families**. DNA sequences of all selected plant species were aligned with the shortest available PPO sequence (PPO1/2 at 1413 bp). These sequences included the conserved tyrosinase domain. Ln Likelihood = -22213.8963; p < 0.01. Species names and PPO annotation were abbreviated for convenience. *Lycopersicon esculentum *Le PPOA/A' [GenBank:Z12833], Le PPOB [GenBank:Z12834], Le PPOC [GenBank:Z12835], Le PPOD [GenBank:Z12836], Le PPOE [GenBank:Z12837, Le PPOF [GenBank:Z12838]; *Medicago sativa *Ms PPO [GenBank:AY283062]; *M. truncatula *MtPPO1 and Mt PPO2 [GenBank:AC157507.2]; *Nicotiana tabacum *Nt PPO [GenBank:Y12501]; *Solanum tuberosum *St PPO32 [GenBank:U22921], St PPO33 [GenBank:U22922]; *Trifolium pratense *Tp PPO2 [GenBank:AY017303], Tp PPO3 [GenBank:AY017304], Tp PPO4 [GenBank:EF183483.1], Tp PPO5 [GenBank:EF183484.1].

A phylogenetic analysis of DNA coding sequences confirmed sequence similarities within species, and showed differences between PPO sequences from Solanaceous and leguminous species (Figure 7; p < 0.01). Bootstrapping exercises were applied to the datasets to measure how consistently the data support given taxon bipartitions. All the tree branches support values generated in this study have high support values (>50%) and therefore provide uniform support.

Sequences from different PPO genes of the Solanaceous species, *Solanum tuberosum *and *Lycopersicon esculentum *(*Solanum lycopersicon*), showed a high level of similarity between, as well as within, species (Figure 7). Within the legumes, PPO sequence from *Medicago sativa *was more similar to the two *M. truncatula *and *Vicia faba *sequences than to the seven T. *pratense *sequences. In *T. pratense *PPO1/2, PPO1/4 and PPO1/5 exhibited the highest similarity, followed by PPO3 and PPO5 (Figure 7).

## Discussion

### Characteristics of BAC library

The genome size of *T. pratense *was previously estimated as 440 Mb [20] and 637 Mb [19]. The average BAC insert size was estimated as 135 Kb therefore, the predicted genome coverage of the library was 6–8 ×. This library complements two existing red clover libraries with smaller average insert sizes at 80 and 108 Kb [20]. A library with a larger insert size offers an advantage in reducing the number of clones required for adequate coverage of the genome. This will also simplify screening the generation of BAC contigs as demonstrated in this study and physical mapping.

### PPO copy number

Numbers of BAC clones in the library containing PPO1, PPO2, PPO4 and PPO5 varied from four to ≥ 28 (Table 1). Between five and six copies of PPO2, PPO4 and PPO5 were detected in the library, suggesting that these genes are present as single copies in the red clover genome. Both PPO3 and PPO5 were detected in genotypes of three red clover cultivars, suggesting separate genes. The high homology of their 3' flanking sequences may indicate a duplication event. However, PPO3 was not identified in the BAC library. This may have resulted from an uneven distribution of restriction enzyme recognition sites throughout the genome [21]. Regions with low numbers of restriction sites may be under-represented, while regions with higher number of restriction sites may create fragments smaller than the cut off fragment size, which in our case was <90 Kb.

By contrast, a minimum of 28 potential BAC clones containing PPO1 were identified in the library, indicating multiple copies. Sequencing indicated three PPO1 variants: PPO1/2, PPO1/4 and PPO1/5, (Figure 3). PPO1/2 was detected in four BAC clones indicating a single copy in the genome, whilst PPO1/4 was detected in at least 26 BAC clones suggesting either multiple copies or an over-representation of this gene in the BAC library. The latter is most likely since BAC ends of both BAC clones that contain PPO 1/4 alone map onto BAC 212 G7, indicating that the solitary PPO1/4 gene actually resides within the PPO cluster. PPO1/5 was detected in a total of nine BAC clones, representing one or possibly two predicted copies. Four PPO1/5 genes were sequenced; while three were identical, the fourth had near identical homology in both gene and flanking sequences and a 100 bp out of 1.7 Kb deletion in the 3' flanking region, suggesting allelic variation.

### PPO family of genes and genome structure

The results presented in this manuscript indicate that there are five distinct paralogous genes in the red clover multigene PPO family: PPO1–PPO5. The BAC library has yielded full length gene sequences and upstream regulatory regions for two new PPO genes, PPO4 and PPO5, and for two variants of PPO1, PPO1/5 and PPO1/4. There were no introns identified in the newly identified red clover PPO genes and variants. This was in agreement with results reported previously for PPO in other dicotyledonous species, including hybrid poplar [22], potato [23], tomato [17] and red clover [15], and as predicted from *M. truncatula *genomic sequences [GenBank: AC157507.2], but is in contrast to PPO genes identified in monocotyledonous species, such as pineapple [24], wheat [GenBank: EF070147 to GenBank: EF070150[25]], rice [GenBank: AP008210], *Lolium perenne *[GenBank: FJ587212] and *Festuca pratense *[GenBank: FJ587213].

The occurrence of multiple PPOs on single BAC clones and the putative alignment of four BAC clones with six distinct PPO genes on an estimated 190–510 Kb fragment is strong evidence for a PPO gene cluster in *T. pratense *(Figure 5). The order and presence of three PPO genes were confirmed by sequencing a 156,267 bp BAC clone, 212 G7. Similar PPO clusters were previously reported in tomato [16] where seven genes were reported as clustered over 165 Kb and detected both in *M. truncatula*, where there are two PPO genes present in 8 Kb of sequence [GenBank: AC157507.2] and in rice, where two active PPO genes and a redundant PPO pseudogene (Figure 6; [GenBank: AP008210.1]) are present in 30 Kb of sequence; rice PPO2 also contains a 11.3 Kb retrotransposon-like insert exhibiting 94% homology with a gypsy-type retrotransposon in rice [GenBank: AB030283] [26]. Retrotransposon insertion into the maize *waxy *gene does not appear to have impaired protein coding ability [27].

No other genes were identified in the vicinity of the red clover PPO cluster, although retrotransposons and regions of homology with *M. truncatula *and *Lotus japonicus *genomic sequence were found on the sequenced BAC 212 G7. Retrotransposons are implicated in gene duplication, altering patterns of gene expression and generating new functions in legumes and maize [27-29].

Clustering of duplicated genes is a well-established phenomenon in plants. This could influence gene function and facilitate co-ordinated expression, and, in duplicated genes, such as PPO genes, minor changes in position may allow subtle changes in regulation, which may benefit the plant under new selection regimes by creating novel tissue-specific or environmentally induced expression.

### Evolutionary implications

Gene clustering and the occurrence of paralogous sequences in the PPO gene family can hint at underlying gene evolution and function mechanisms. For example, paralogous genes are widely recognised and expected to have diverged by a minimum of 10% over time [30]. Four of the five PPO genes have diverged by 10% or more at the cDNA or amino acid levels (Figure 4), whereas PPO3 [15] and the newly sequenced PPO5 share 94% identity. This is substantially higher than the 80–90% identity expected for ancient paralogues. Nearly identical paralogues (NIPs) have been defined as paralogous genes that exhibit ≥ 98% identity [30]. Such NIPS are claimed to allow differential expression within the gene family and increase plasticity of the transcriptome [30]. In red clover, variants of PPO1 may be considered as NIPs: PPO1/2, PPO1/5 and PPO1/4 exhibit more than 98% identity.

The different PPOs, including the three NIPs of PPO1, have presumably arisen due to partial genome duplication, the extent of divergence relating to the timing of the duplication event(s). PPO gene sequences vary considerably, forming clear phylogenetic groups for higher plants, vertebrates, fungi and bacteria [31]. DNA sequences show high homology within species and within families, such as Solanaceae (*Solanum, Lycopersicon *and *Nicotiana *species) and Fabaceae (*Vicia*, *Trifolium *and *Medicago *species) (Figure 7).

The divergence of PPO genes within red clover is similar to that observed within other plant species. For example, the two PPO genes identified in *M. truncatula *have 90% identity [GenBank: AC157507.2] whereas the seven clustered genes [GenBank: Z12833, Z12834, Z12835, Z12836, Z12837, Z12838] in the tomato PPO family have between 73 and 97% identity [17].

Red clover possesses a large, functional PPO gene family (Figure 7). While PPO enzymes are expressed constitutively in aerial and root tissues of *T. pratense*, PPO enzymes only exist in a latent or inactive form in leaf tissue of both *T. repens *(unpublished data) and *V. faba *[14]. By contrast, PPO activity is not detected in other agronomically important forage legumes, such as *Medicago sativa *(alfalfa) and *Lotus corniculatus *(birdsfoot trefoil), or in the model species *M. truncatula *and *L. japonicus *(unpublished data). At least one PPO gene is present in *M. sativa*, and two in *M. truncatula *yet, to date, no ESTs have been reported for either species. It is possible that conditions have not yet been determined that elicit PPO gene expression in these species, but the apparent lack of PPO transcript concurs with failure to detect PPO enzyme activity in tissues of either species.

These observations raise questions about the evolution of PPO genes both within *T. pratense *and between *T. pratense *and its close relatives. Phylogenetic trees of divergence of *T. pratense *PPO DNA sequences (Figure 7) confirm the level of identity of red clover PPO at the genetic level, with PPO1 NIPs being most similar and probably, therefore, most recently diverged [22,32].

Diversification of plant genomes is powered in part by gene duplication, which can result in new gene functions [33]. Such gene duplication may occur by creation of polyploids, by segmental duplication or duplication in tandem arrays resulting in the production of gene clusters. Positive selection is believed to play a crucial role in the retention of such duplicated genes [33] but the effect of positive selection on tandem arrays or clusters of genes is not clear [18]. Over time, individual PPO genes and PPO clusters may have originated, duplicated and subsequently been lost, their function governed by mutations in regulatory elements. A comparison of selected PPO DNA sequences in both red clover and tomato (Figure 7), indicates that such gene duplication has occurred leading to clusters of six or seven similar PPO genes, each with known, different expression patterns.

### PPO localisation and function

The biological effects of PPO appear to be subtle, possibly requiring specific or even multiple triggers for expression *in vivo*. Enhanced localised PPO expression under biotic and abiotic stress provides evidence of its involvement in plant protection in various species, for example, localised PPO expression in leaf abscission zones during drought [34]. A multiple regulatory trigger might explain the prerequisite of plant hardening, by low temperature and low light mimicking autumn conditions, before any difference in susceptibility to *Sclerotinia trifoliorum *was detected between late and medium-late flowering types of red clover [35]. Similarly, differences in survival of low PPO-mutant and wild-type red clover plants only became apparent under multiple, natural infestations [8].

The high degree of homology in active sites of red clover PPO indicates similar enzymic properties. However, differences do occur in localisation of PPO enzyme activity and specific PPO gene expression in both red clover [15] and tomato [7,16,17], suggesting significant differences in their regulatory elements; this is supported by observed differences in sequenced promoter regions of four red clover PPO genes. Red clover PPO genes are differentially expressed in aerial tissues and root tissues [15] conferring the potential for enhanced or localised expression following differing abiotic and biotic stimuli.

## Conclusion

The red clover BAC library has yielded novel full-length gene sequences of PPO4, PPO5 and the PPO1 NIP PPO1/4, which will be used in functional studies involving techniques such as RNAi, and the PPO promoter sequences will be used for localisation studies using promoter::reporter gene fusions. It has also revealed recent gene duplication events in the form of NIPs and evidence of gene clustering. The BAC library will provide a useful tool for the map-based cloning of target QTL, physical mapping, genome structure analyses and the alignment of specific regions of the *T. pratense *genome with its close relatives the model legume, *M. truncatula*, and other legume species such as alfalfa, revealing any genomic changes or divergence at these sites. The high degree of synteny between *T. pratense *and *T. repens *with both *M. truncatula *and *M. sativa *[20,36] will allow comparative mapping between model and agronomically important legumes.

## Methods

Construction of the red clover BAC library was based on procedures described previously [37].

### Isolation of high molecular weight genomic DNA

High molecular weight (HMW) DNA was isolated from a single genotype of diploid *T. pratense *cultivar Milvus (2n = 2x = 14). The plants were maintained in darkness for 42 h prior to harvesting a total of 21.9 g leaf tissue. The leaf tissue was frozen and stored at -80°C. Leaf tissue was ground in liquid nitrogen and nuclei isolated [38]. The nuclei were embedded in agarose plugs and, before digestion, the HMW DNA was subjected to a pre-electrophoresis step on a 1% (w/v) agarose (Sigma, St Louis, MO, USA) gel using a CHEF-DR II PFGE apparatus (Bio-Rad, Hercules, CA, USA) [39,40].

### Partial digestion and size selection of digested DNA

The entire library was generated from a single size selection experiment. *T. pratense *DNA was partially digested using *Hind*III (Roche, Mannheim, Germany) and separated in a single step, on a 1% (w/v) pulse field certified agarose gel, by PFGE at 5.2 V cm^-1 ^for 16 h with a linear pulse ramp from 0.5–40 s using a CHEF-DR II apparatus (BioRad). Partial digestion was performed using a low enzyme concentration (0.5 U/plug) at 37°C for 1 h, which in preliminary studies resulted in a smear of DNA between 160 Kb and 90 Kb but no significant DNA below this on the gel.

Following electrophoresis, the flanking regions of the gel containing HMW DNA ladder (lambda ladder PFG marker; NEB, Beverly, MA) were stained with ethidium bromide and marked under UV so that alignment with the unstained gel allowed the selection of one gel slice in the range of 100–150 Kb. This gel slice was then excised and the partially digested genomic DNA recovered by dialysis [39].

### Ligation and transformation

The partially digested DNA was ligated with *Hind*III-digested pIndigoBAC-5 vector (Epicentre Biotechnologies, Madison, WI, USA) using a predicted vector/insert molar ratio of between 5:1 and 10:1. Ligations were carried out in 1× T4 DNA ligase buffer at 14°C overnight using 1 Weiss unit of T4 DNA ligase (Roche) per 50 μl of ligation buffer. The ligation reaction was drop dialysed and 1 μl of the ligation product was transformed into 20 μl of *Escherichia coli *ElectroMAX DH10B competent cells (Invitrogen, Carlsbad, CA, USA) by electroporation (GenePulser II; Bio-Rad). Transformed cells were allowed to recover in 1 ml SOC media (2% w/v bacto tryptone, 0.5% w/v bacto yeast extract, 10 mM NaCl, 2.5 mM KCl, 10 mM MgCl_2_, 20 mM glucose, pH 7.0) at 37°C for 45 min with shaking at 180 rpm, and plated out on LB plates containing 12.5 μg ml^-1 ^of chloramphenicol and incubated at 37°C overnight [37,41].

### Picking and storing

BAC colonies were picked in duplicate into 200 μl of Freezing Broth (LB, 36 mM K_2_HPO_4_, 13.2 mM KH_2_PO_4_, 1.7 mM Na Citrate, 0.4 mM MgSO_4_, 6.8 mM (NH_4_)_2_SO_4_, 4.4% v/v Glycerol, 12.5 μg ml^-1 ^chloramphenicol) in 96-well microtitre plates using a GloPix robot (Genetix, New Milton, Hampshire, UK). Following overnight incubation at 37°C, plates were stored at -80°C. A total of 26,016 BAC clones were picked into 271 96-well plates.

### Determination of insert size of BAC clones

A total of 58 BAC clones chosen at random were selected, cultured overnight and insert size determined. Following *Not*I restriction digestion, isolated DNA was separated by PFGE in the presence of molecular weight markers in order to estimate the average insert size of the cloned DNA.

### Pooling of BAC library for PCR-based screening

The library was replicated in microtitre plates and plate cultures pooled in such a way as to enable a PCR-based screen of the library [37]. A total of 271 microtitre plates of clones were used as the basis for the screen. Each plate was represented in three superpools so that, following DNA extraction, a PCR screen of 147 DNA superpools would generate three positive amplifications per positive BAC colony. Once the superpools had been created, 50 ml plastic tubes containing the pooled cultures from up to seven plates were centrifuged at 5000 rpm in a model 5403 centrifuge (Eppendorf, Hamburg, Germany). The supernatants were discarded and the pellets frozen at -80°C. BAC DNA was isolated from the stored pellets using an alkaline lysis method, which included RNase in the resuspension buffer. Superpool DNA was precipitated using isopropanol, the pellet washed with 70% ethanol and resuspended in TE.

### PCR-based screen of the BAC library

The DNA superpools of the BAC library were screened using PCR primers for amplification of individual genes. PCR primers were designed from sequences of five *T. pratense *PPO genes: PPO1 [GenBank: AY017302.1], PPO2 [GenBank: AY017303.1] and PPO3 [GenBank: AY017304.1] and from partial PPO4 and PPO5 sequences identified in this study. Following the initial BAC library screen, PCR primer pairs were also designed for PPO1 variants PPO1/2, PPO1/4 and PPO1/5 (Table 2).

**Table 2:** PCR product size and PCR primer pairs used to amplify PPO genes

Gene	PCR product (bp)	Forward	Reverse
PPO1	401	CGGCGGTGACACACCTTTC	GGAAGGGCGTTGTGCTGATG

PPO2	229	CAACAAGAAGAAGGAGAAGAAG	AGCACCACCACGAGAAGAAT

PPO4	150	ACGAAGGTGGCGTAGATGAC	CATTTCCATGGTGAGCGTAA

PPO5	391	GCAAATCTAAGGAGGATCCTACCG	AGTCTCTAGCCAATCATCGTC

PPO1/5	200	GGAATGTCAAAATTAGTGGC	ACATTGATTACAATATATTCC

PPO1/2	140	ATCGTTGACCTAAACTATAACAG	GAAAGGTGTGTCACCGCC

PPO1/4	200	ATAGAAAACCAAAGCACC	ATTTTCATATCATCTGGTAAC

PPO deg	764–771	GCCMYTRAWCTCATGARAGC	CTCATCATARAARAGAAA

PPO deg – PPO degenerate primers

### Isolation and identification of genes in PPO family

PPO fragments were generated by PCR from red clover genomic DNA (cultivar Milvus) with degenerate primers based on regions of homology to PPO genes from *T. pratense *and *Vicia faba *(PPO deg; Table 2). PCR amplification products were visualised on an agarose gel, excised, purified and cloned into *E. coli *(Invitrogen Topo TA Cloning^® ^kit with pCR^®^2.1 TOPO^® ^vector and TOP 10 One Shot^® ^Cells). Inserts were sequenced and a number of PPO genes were detected, including two novel genes designated PPO4 and PPO5. PPO4 and PPO5 were isolated from the BAC library using specific primers (Table 2) designed to specifically amplify individual genes. Three variants of PPO1 (PPO1/2, PPO1/4 and PPO1/5) were also sequenced These PPO1 variants were designated codes according to their juxtaposition with other PPO genes on BAC clones: PPO1/2, PPO1/4 and PPO1/5 were initially detected on BAC clones along with PPO2, PPO4 or PPO5, respectively. Once identified, selected PCR-positive BACs for each gene were sequenced directly using specific primers (Table 2) and an ABI prism 3100 DNA analyser (Applied Biosystems, Warrington, UK). BAC walking was used to generate full length gene and upstream promoter sequences of PPO genes.

### Sequencing and *in-silico *analysis

Sequencing of PCR products and BAC clone plasmids was carried out using an ABI-3100 Genetic Analyser (Applied Biosystems) using fluorescent dye terminators. A BAC clone (designated 212 G7) harbouring genes PPO1/5, PPO5 and PPO1/4 was fully sequenced on a Roche 454 GS-FLX™ system, giving an average of 30,000 reads or 6 Mb of data (Cogenics).

Sequences were assembled and further analysed using Vector NTI software and NCBI/BLAST and FASTA programs. Sequences were compared to public DNA, EST and protein (NCBI) databases and existing red clover PPO gene sequences to confirm their identity.

PPO DNA sequences were aligned in Vector NTI Advance 10, based on ClustalW algorithm, and displayed in PHYLIP 3.67. For valid comparisons, DNA sequences of all selected plant species were aligned with the shortest available PPO sequence (PPO1/2; 1413 bp) and truncated in line with this sequence: the truncated sequences contain the conserved domain. DNA sequence data were analysed statistically by Maximum Likelihood Method and the phylogeny tree was generated using PHYLIP http://evolution.genetics.washington.edu/phylip.html[42].

### Accession numbers of new red clover and grass PPO sequences

Identified PPO genes sequences were submitted to GenBank: *Trifolium pratense *PPO4 [GenBank: EF183483.1], PPO5 [GenBank: EF183484.1], PPO1/4 [GenBank: FJ587214]; *Lolium perenne *[GenBank: FJ587212]; *Festuca pratense *[GenBank: FJ587213].

## Authors' contributions

AW conceived the study, analysed the PPO sequences and jointly wrote the manuscript. SH together with AT created the BAC library and SH helped to draft the manuscript. KF participated in the design and construction of the BAC library and critically evaluated the manuscript. ID participated in the design of the BAC library and critically evaluated the manuscript. KJW conceived the study, analysed BAC sequences, created the phylogenetic tree and jointly wrote the manuscript.
